# A neglected problem in the utilization of free anterolateral thigh flap toward reconstructing complicated wounds of extremities: the obliteration of deep dead space

**DOI:** 10.1186/s13018-020-01914-0

**Published:** 2020-10-21

**Authors:** Gao-hong Ren, Da-yong Xiang, Xiao-hu Wu, Yun-biao Chen, Runguang Li

**Affiliations:** 1 Division of Orthopaedics and Traumatology, Department of Orthopaedics, Nanfang Hospital, Southern Medical University, Guangzhou, 510515 China; 2 Guangdong Provincial Key Laboratory of Bone and Cartilage Regenerative Medicine, Nanfang Hospital, Southern Medical University, Guangzhou, 510515 China; 3 Department of Orthopedics, The Third Affiliated Hospital of Southern Medical University, Guangzhou, 510610 China; 4 Orthopaedic Hospital of Guangdong Province, Guangzhou, 510610 China; 5 Academy of Orthopaedics, Guangzhou, 510610 Guangdong Province China; 6 Department of Orthopedics, Linzhi people’s hospital, Linzhi, 860000 China

**Keywords:** Chimeric flap, Anterolateral thigh perforator flap, Complex wounds, Wound reconstruction, Deep dead space

## Abstract

**Background:**

Deep dead space may be thought as an independent risk factor of the poor infection control after flap reconstruction in complex limb wounds. But it can be easily neglected. The conventional skin flap and musculocutaneous flap are difficult to obliterate the deep dead space in irregular shape effectively. It was investigated that the clinical application of chimeric anterolateral thigh perforator flap in the treatment of complex wounds complicated with deep dead space of the extremities in the paper.

**Methods:**

Fifty-six cases complicated with deep dead space wounds were registered in group. Following thorough debridement and treatment with VSD, the granulation tissues grew with well-controlled infection. And then the chimeric anterolateral thigh perforator flap was used to obliterate the deep dead space and repair the wounds. The postoperative flap survival and infection conditions were evaluated.

**Results:**

Overall, the infection was effectively controlled, without persistent exudation or sinus tract formation after wound healing. While 5 cases lost to follow-up, the remaining 51 cases were followed up until 15 months on average. Generally, the affected extremities recovered satisfactorily with normal appearances and texture of the flaps, along with normal functions. Importantly, no recurrence of infection was observed.

**Conclusion:**

During the grafting of chimeric perforator flap pedicled with lateral thigh muscle flap, the muscle flap is recommended to obliterate the deep dead space while the skin flap is being used to cover the wound. The combination of these two technologies performed well in the repair and reconstruction of the complex wounds of the extremities, possessing potential for broader clinical application.

## Background

Microsurgery is an effective treatment toward various types of wounds in the extremities caused by high-energy trauma in clinical settings. High clinical efficacy has been reported in a large number of studies. Among these studies, free flaps were recommended to be used in the treatment of extensive soft-tissue defects or complex wounds, while patients with poor surrounding soft tissue could not accept the local pedicled flap. Since its first description by Song et al. [1] in 1984, the anterolateral thigh flap has been considered to be an universal and preferential free flap [2, 3] for the reconstruction of limb wounds, owing to its constant anatomical position of nutrient vessels, long vascular pedicle, thick vascular diameter, and relatively concealed donor site. Our group previously utilized the anterolateral thigh flap to reconstruct various types of wounds to achieve satisfactory outcomes [4, 5]. However, repeated exudation and persistent infection surrounding the postoperative flaps in partial complex wounds occurred during the treatment process. Particularly, local deep dead space was constantly observed during the second debridement and a large quantity of inflammatory tissues was noted. We believe that these wounds complicated with deep dead space may be an independent risk factor of the poor infection control after flap reconstruction, and the management of deep dead space plays an important role throughout the reconstruction of complex limb wounds.

After multiple times of debridement of deep tissue injuries, related to the post-traumatic ischemic necrosis and the limb infection, some patients experience wounds with residual deep dead space [6]. The three-dimensional feature of the residual deep dead space is associated with the poor blood supply, unsuccessful effusion drainage, and deficient granulation growth. These factors may provoke or aggravate the infection that requires further debridement. Also, the deep dead space may be enlarged after debridement, bringing challenges to the reconstruction in clinical settings in a vicious cycle pattern. To prevent this pattern, it is essential to obliterate the deep dead space with live tissues that cover the wound and improves blood supply, for the promotion of the growth and healing of the tissue around the cavity. According to previous reports, conventional flap transplantation can only cover the superficial wounds [5, 7, 8]. Despite the advantages such as excellent appearance and mild injury induction of the perforator, it fails to obliterate the deep dead space caused by trauma. Conventional musculocutaneous flap is formed by the musculocutaneous perforating branch from the vascular pedicle. The skin flap and muscle flap receive blood supply from the same perforating branch, thus making it hard to separate them. The partial sliding does not fulfill the requirements of flexibilities to effectively obliterate the deep dead space. Regarding biomaterials, bone cement and negative pressure sponge for vacuum sealing drainage (VSD) have been commonly applied as temporary filling materials to obliterate the deep dead space [9–11], due to the lack of biological activities. With the development of flap grafting technique, especially the broader adaption of chimeric perforator flap, other tissue flaps are incorporated when incising the skin flap. Specifically, the skin flap is responsible to cover the wounds, while the other tissue flaps, such as muscle flap, are employed to obliterate the deep dead space [12]. To better meet the clinical requirements, we used the chimeric anterolateral thigh perforator flap (chimeric perforator flap pedicled with descending branch of the lateral circumflex femoral artery and lateral thigh muscle flap) to treat wounds complicated with deep dead space of the extremities with slight modification from previous reconstruction methods. In this study, clinical data of 56 cases admitted to our hospital from January 2014 to December 2018 were collected and retrospectively analyzed to evaluate the clinical efficacy of this modified technique.

## Materials and methods

### Inclusion and exclusion criteria

Inclusion criteria were as follows: all of these patients experienced severe extremity injuries accompanied by varying levels of tissue necrosis, tissue infection, and deep-tissue exposure. The residual deep dead space was observed after debridement. It is suitable to use the anterolateral thigh flap to repair the wound. Exclusion criteria were as follows: patients unsuitable or unable to tolerate microsurgery.

### Baseline data

Fifty-six cases were enrolled, aged 3-72 years with an average of 33.4 years. The wound sized were from 8 cm × 6 cm to 30 cm × 15 cm. Patient details are shown in Table 1.

**Table 1:** The detailed injury information of the patients

		Cases
Total		56
	Male	38
	Female	18
Causes of injury	Vehicle or motorcycle bruise injuries	34
	Falling related injuries	12
	Heavy objects crush injuries	10
Exposure form of deep tissue	Formed by tendon and muscle exposure	22
	Formed by bone and joint exposure	29
	Exposure of internal fixators such as plates	5
Complicated with the ipsilateral fractures		41
Complicated with fractures in other bone parts or systematic injuries		21

### Treatment methods

#### Preparation prior to wound reconstruction

According to the conditions of the wound contamination or infection prior to wound reconstruction, 56 cases were treated with debridement and VSD as previously described [4, 5, 13]. Following the wound cleaning, the chimeric perforator flap pedicled with descending branch of lateral circumflex femoral artery and lateral thigh muscle flap was applied to obliterate the deep dead space and reconstruct the wounds.

#### Operation process

**Fig. 1:**
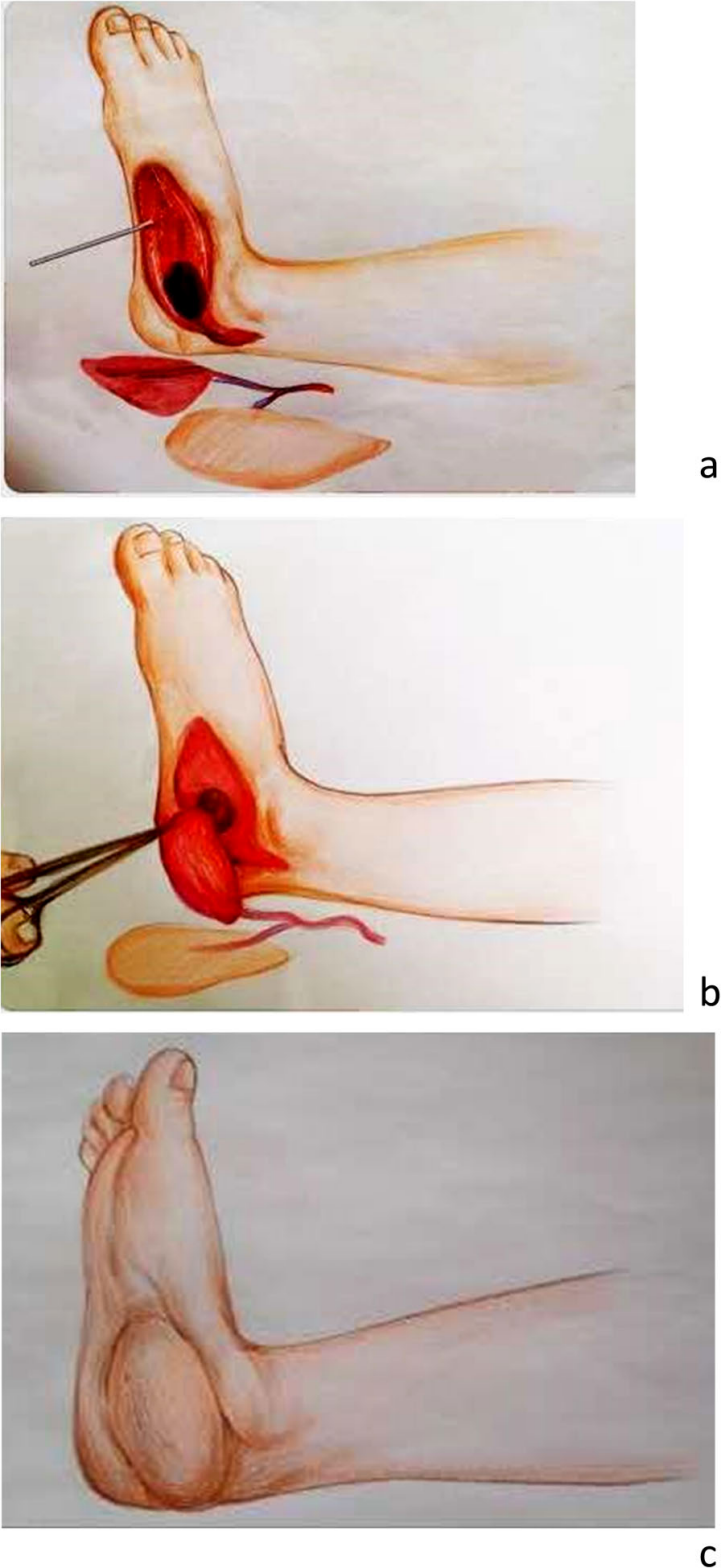
Diagram illustrated the design process of chimeric anterolateral thigh perforator flap for the repair of complex wounds complicated with deep dead space of the extremity (**a-c**)

General or combined spinal-epidural anesthesia was given. The patients were required to lay in a supine position. Preoperative Doppler ultrasound was performed to locate the descending branch of the lateral circumflex femoral artery. The microsurgical operations of 65 cases were performed by two different surgeons, all of whom had received systematic microsurgical training and had at least 5 years of independent microsurgical experience. According to the methods of Luo S et al. [14] and Lee YC et al. [15], we designed the flap and exposed the perforating vessels of the flap. According to the size of deep dead space and the requirements of tissue reconstruction, one or more leaves of muscle flaps in an appropriate size and length were harvested with the descending branch of lateral circumflex femoral artery as the pedicle. The chimeric flap was grafted to the recipient site after the incising of the pedicle. With the adjustment of the distance between the chimeric flap and the vascular pedicle, the lateral thigh muscle flap was utilized to obliterate the deep dead space and the perforator flap was used to cover the wound. The descending branch of the lateral circumflex femoral artery and its accompanying vein were anastomosed with the vessels at the recipient site under the microscope (Fig. 1). Specific adjustments were adopted according to the patient’s conditions. For instance, we enlarged the anastomosis sites of blood vessels by anastomosing the vessels of the skin flap with the distal vessels of the muscle flap for 5 cases with vascular separation between the skin flap and muscle flap. In contrast, the vascular pedicles of the lengthened flaps were grafted with the great saphenous vein or the descending branch of the lateral circumflex femoral artery and vein [13, 16], for 6 cases with insufficient length of vascular pedicles.

#### Postoperative management

Conventional microsurgical therapies, such as anti-inflammation, anti spasm, and anti-coagulation, were given as needed. According to preoperative bacterial culture results, appropriate antibiotics were selected for subsequent treatment. The blood supply of the flap and the blood ooze beneath the flap were intensively monitored, and postoperative complications including vascular crises were timely and effectively managed. Based upon the status of wound and deep tissue reconstruction, active and passive rehabilitation exercises were actively carried out as soon as possible after the operation. Moreover, the survival and infection of flaps were evaluated postoperatively.

## Results

Among 56 cases, 2 cases developed vascular crisis, which was alleviated with timely vascular exploration. One case had excessive bleeding after flap grafting, which was resolved with surgical exploration. Three cases experienced partial necrosis of the flaps and, leaving residual local wound, which were completely eliminated by secondary skin grafting in one case and by proactive dressing changes in the other 2 cases. Overall, the infection was well controlled. No persistent exudation or sinus tract formation occurred after wound healing. Except 5 cases lost to follow-up, the remaining 51 cases were followed up until 15 months on average (ranged 9-24 months). Generally, the affected extremities recovered satisfactorily with normal appearances and texture of the flaps, along with normal functions. Importantly, no recurrence of infection was observed.

**Fig. 2:**
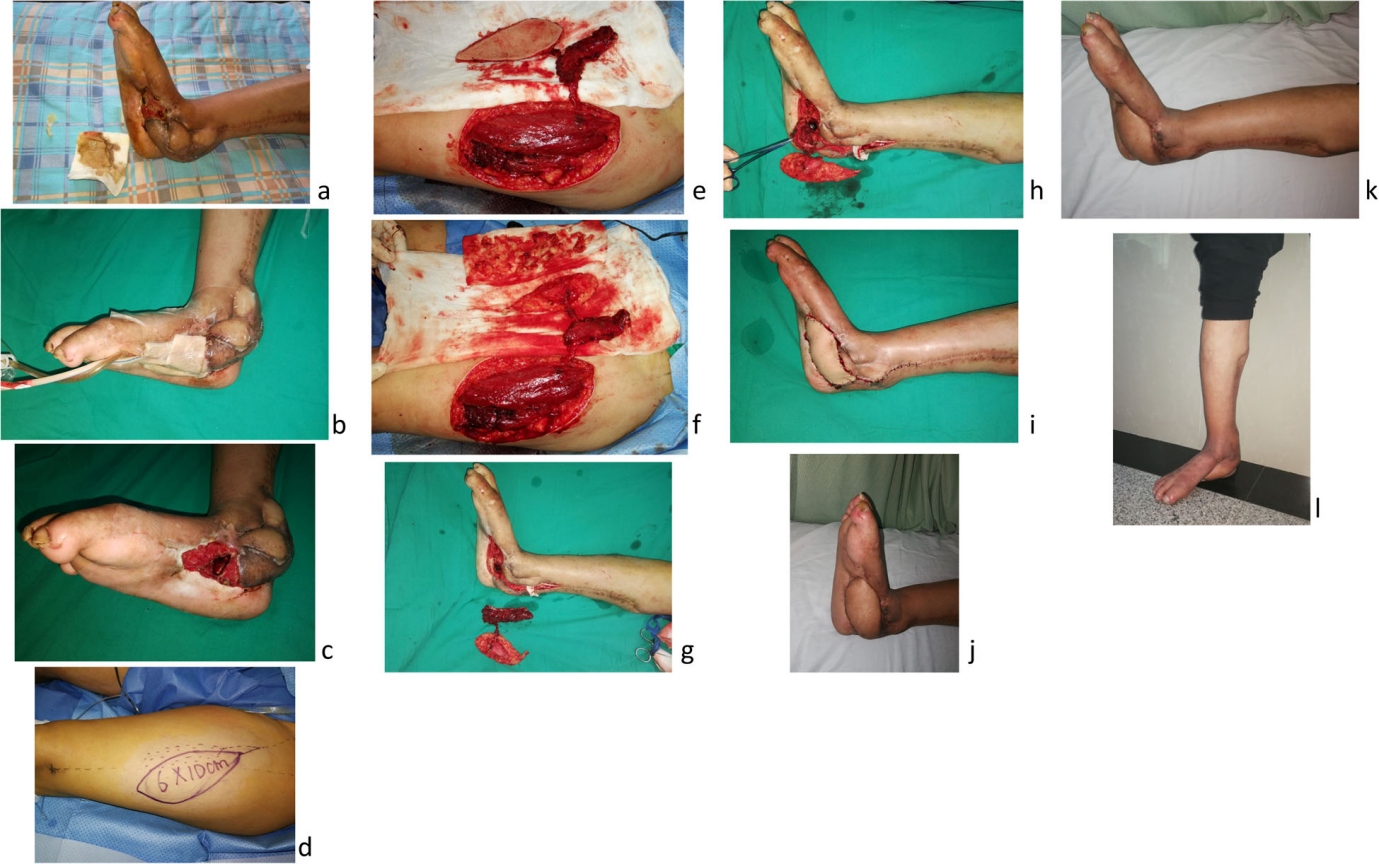
Case 1. A male patient, 49 years old, was admitted to our hospital due to postoperative deformity and infection of the right foot for over 6 months. Upon admission, a large quantity of purulent secretion was noted in the medial wounds of the right foot, accompanied with sinus tract formation in the right calcaneus, stiffness, and deformity of the ankle (**a**). After admission, thorough debridement combined with VSD was performed (**b**). At one week after the VSD removal, granulation tissues surrounding the wounds grew well (**c**). The chimeric anterolateral thigh perforator flap was designed and harvested, and the perforator flap was thinned (**d-f**). The muscle flap was utilized to obliterate the dead space of calcaneal defects (**g**, **h**). The skin flap was used for wound coverage and the blood supply was excellent after vascular anastomosis (**i**). During postoperative, twelve months follow-up, the foot infection was effectively controlled, with esthetic appearance of the skin flap (**j**, **k**), and the weight-bearing and walking functions of the foot was restored (**l**)

**Fig. 3:**
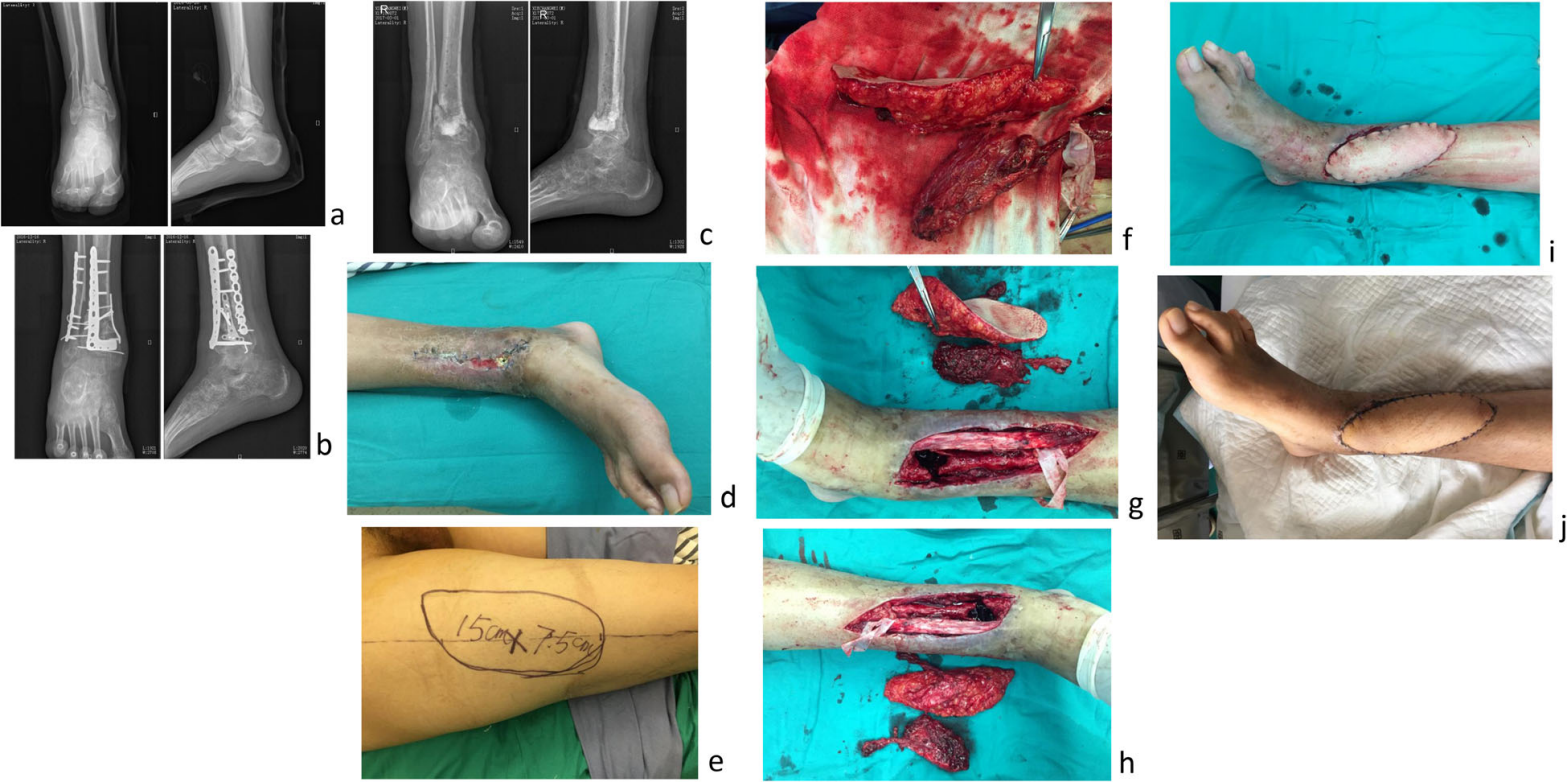
Case 2. A 24-year-old patient suffered from bilateral pilon fractures caused by high falling, open fracture (Gustilo type II) on the right side. After admission, thorough debridement was given, followed by internal fixation of the tibial and fibulal fractures (**a**, **b**), wound healing was poor, secondary infection occurred, and the internal fixation plate was exposed. The internal fixation plate was removed and filled with bone meal (**c**), but the wound infection remained uncontrolled (**d**). The chimeric anterolateral thigh perforator flap was designed and excised (**e**, **f**). The muscle flap was employed to obliterate the dead space (**g**, **h**), the skin flap was used for wound coverage (**i**). Postoperative infection was well controlled and the flaps were normal in appearance and texture (**j**)

**Fig. 4:**
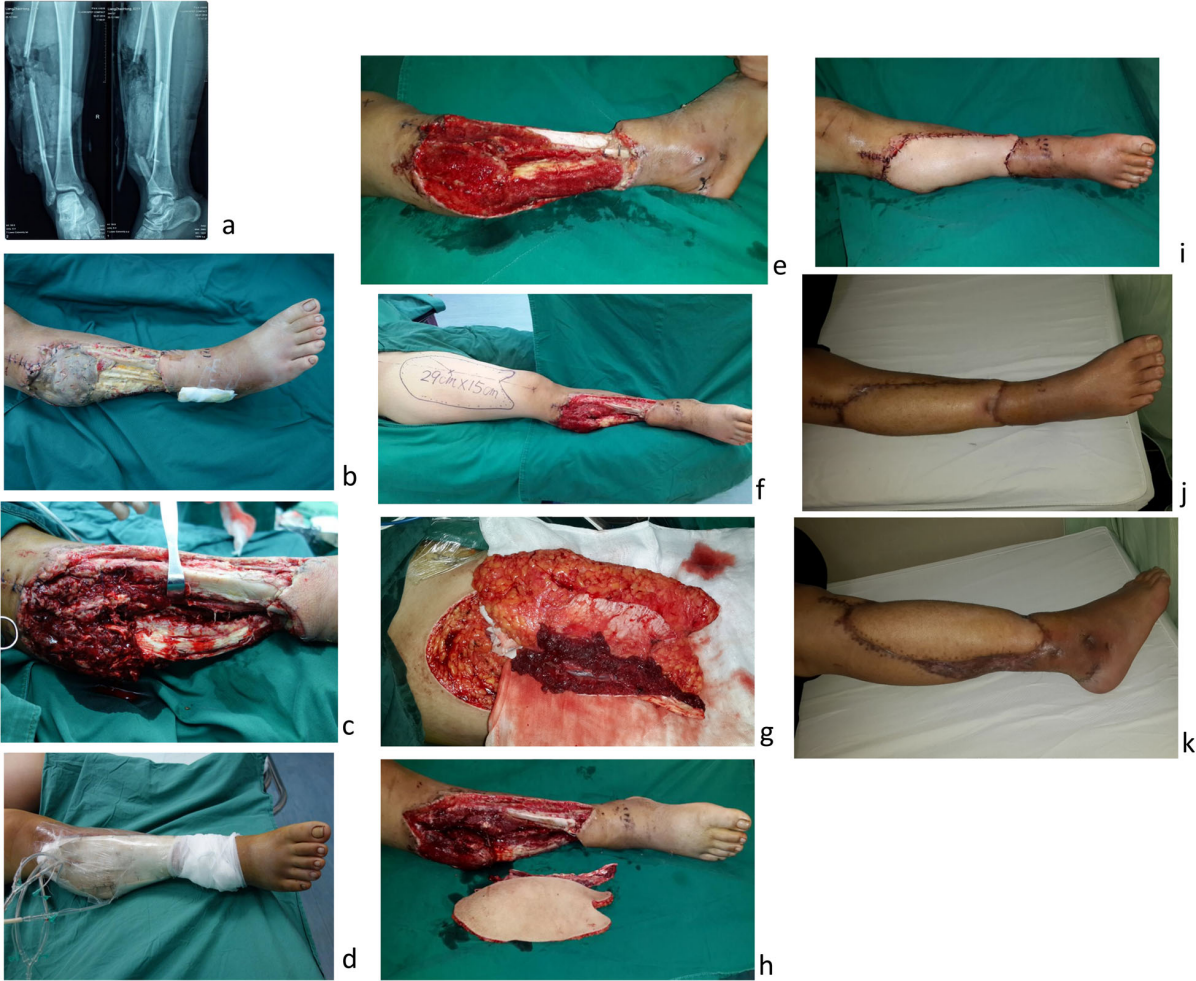
Case 3. A male patient, 22 years old, was transferred from a local hospital due to postoperative infection for 2 weeks caused by traffic injury of the right leg. Upon admission, extensive necrosis of the lateral soft tissues of the middle and lower right leg observed, accompanied with fracture of the middle fibula, free and exposed bones, and purulent secretions in the wounds (**a**, **b**). Upon admission, thorough debridement combined with VSD (twice) was performed, and granulation tissues surrounding the wounds grew well (**c**-**e**). The chimeric anterolateral thigh perforator flap was designed and harvested (**f**, **g**). The muscle flap was utilized to obliterate the dead space of the lateral leg, and the skin flap was used to cover the wound. The blood supply was excellent after vascular anastomosis (**h**, **i**). The flap was normal in appearance (**j**, **k**). During postoperative follow-up for 12 months, no obvious sinus tract formation was observed and the infection was properly controlled

Typical cases were shown in Figs. 2, 3, and 4.

## Discussion

Soft-tissue defects of extremities complicated with deep dead space caused by high energy injury bring challenges to the wound reconstruction in the clinical setting. Tissue defects, with poor local blood circulation, along with unsuccessful drainage often induce infections. In addition, some vital tissues and organs, such as bones, large blood vessels, nerves, muscles, tendons at a special site may exist in the deep dead space. Therefore, we are facing a dilemma, since thorough debridement could lead to serious functional loss of these tissues or organs, whereas incomplete debridement could cause infections. Furthermore, some special deep dead spaces, which are associated with the articular cavity, internal fixation, pressure ulcer, or diabetes mellitus, further increase the difficulty of wound reconstruction. The conventional skin flap and musculocutaneous flap are difficult to obliterate the deep dead space in irregular shape effectively. To overcome the challenges in these types of reconstructions associated with the three-dimensional requirement of the flap, the elimination of the deep dead space, as well as the coverage of the very large soft-tissue defect in a single procedure with primary closure of the donor site are needed [17].

The chimeric perforator flap is a special form of perforator flap, which refers to two or more different types of independent tissue flaps (such as skin, fascia, muscle, and bone) harvested in the same vascular supply area. At least one perforator flap is included in these independent tissue flaps, and the nutrient vessels originate from the same primary blood vessels. Anastomosing a group of vascular pedicle (primary blood vessels) can reconstruct the blood circulation of multiple independent tissue flaps at the same time [17, 18]. In this study, we used the chimeric perforator flap pedicled with descending branch of lateral circumflex femoral artery, which is a special type of perforator flap [19]. In addition to the merits of traditional anterolateral thigh flap, it holds advantages as described in the following. The blood supply of the multiple independent tissue flaps can be simultaneously reconstructed by anastomosing one group of blood vessels. This technique retains the advantages of musculocutaneous flap, such as excellent blood supply and potent anti-infection capability. Both perforator flap and muscle flap have vascular pedicles with a sufficient length. The lateral thigh muscle flap has excellent shaping ability, to effectively obliterate irregular deep dead space. Moreover, the delivery of skin grafting on the exposed muscle flap minimizes the incisional area of skin flap, which is consistent with the concept of “appropriate incision amount” without incising other ineffective tissues while incising the multi-leaf skin flaps and muscle flaps.

Thigh anterolateral muscle is rich, even if the amount of the harvested tissue is larger, also will not cause limb dysfunction. In a study that involves 65 cases that underwent free anterolateral thigh chimeric flap reconstruction of defects in the head and neck regions, Kun Wu et al. [2] reported a new classification concept to divide the anterolateral thigh chimeric perforator flap into 3 types: trunk type (I type, 16.9%), branch type (II type, 69.3%), and bifurcation type (III type, 13.8%). It had certain significance for all kinds of tissue defect repair. In our present study, 5 cases showed vascular variations, in which the skin flap and muscle flap vessels were separated and did not originate from the common junction. To solve this problem, a novel chimeric flap was developed by anastomosing the nutrient vessels of the perforator flaps with the distal vessels of the muscle flaps.

The chimeric perforator flap pedicled with descending branch of lateral circumflex femoral artery is a novel and practical procedure, which achieves three-dimensional reconstruction of the wounds with an expanded application range of flaps for the reconstruction of complex soft-tissue defects of the extremities. There are some previous studies that utilized similar concept but different details. For example, N.A.S. Posch et al. [20] used combined free partial vastus lateralis with anterolateral thigh perforator flap to reconstruct extensive composite defects. Qing L et al. [21] designed individual vastus lateralis muscle-chimeric multi-lobed anterolateral thigh perforator flap to reconstruct complex three-dimensional defects in the extremities. Zheng X, et al. [22] performed single-stage reconstruction and revascularization using a free flow-through chimeric anterolateral thigh perforator flap under emergency.

We reconstruct the limb defects complicated with residual deep dead space in 56 cases and achieved high clinical efficacy in 51 cases with complete post-operational follow-up, while no persistent wound exudation or sinus tract formation observed. Consistently, in the study of adult chronic tibial osteomyelitis cases, Buono P et al. [23] demonstrated that fasciocutaneous and perforator free flaps offer a comparable efficacy to the muscle flaps for infection treatment, with a significantly higher patient satisfaction and esthetic result. To be noted, the cases of extremity defects complicated with the closure of deep dead space were included in our current study, and alternative surgical methods should be combined for those with structural tissue defects. If structural tendon, nerve, and bone tissue defects were observed at the recipient site, the reconstruction of these tissues should be performed using techniques such as free revascularized fibula and iliac crest bone flaps [24] or Ilizarov technique [25], and Mesquelet technique [26]. Postoperative clinical efficacy and limb function of these methods were not to be described here.

In this study, the vascular crisis occurred in 2 cases after operation and extensive bleeding occurred in one case after flap grafting, which requires accurate judgment and precise operation to be dealt with. The main cause of vascular crisis is thrombosis [27], associated with the quality of blood vessels at the recipient site, such as the intimal separation, especially in patients with serious contusion, long-term wound infections, diabetes mellitus, smoking, old age, etc. Consequently, it is essential to dissect and separate the healthy vessels to anastomose with the vessels at the donor site. Because the perforating branch vessels are relatively thin, a certain amount of surrounding tissues should be preserved to avoid distortion, traction, or compression of the perforating branch vessels. Particularly, vascular traction could be caused by extensibility of muscle during the filling of a muscle flap. Moreover, attention should be paid to hemostasis at the recipient wounds, the incised skin flap, and muscle flap, especially for the inflammatory granulation tissues with sufficient blood supply. If intraoperative hemostasis were not performed effectively, the anticoagulant and anticonvulsant drugs were used after operation, and pressure bandage could not be applied to the wound in the recipient area, then massive hemorrhage or hematocele at the wound sites would occur.

## Conclusions

Deep dead space is a problem in the process of limb wound reconstruction that can be easily neglected. The chimeric perforator flap pedicled with a descending branch of lateral circumflex femoral artery can be applied to treat limb wounds complicated with residual deep dead space, which uses muscle flap to effectively obliterate the deep dead space and employs perforator flap to cover the wounds. This technique in our study yields satisfactory clinical efficacy, providing foundations for further clinical application.

## Data Availability

None

## References

[1] Song YG, Chen GZ, Song YL (1984). The free thigh flap: a new free flap concept based on the septocutaneous artery. Br J Plast Surg.

[2] Wu K, Ji T, Cao W, Wu HJ, Ren ZH (2019). Application of a new classification of chimeric anterolateral thigh free flaps. J Craniomaxillofac Surgery.

[3] Zhang Y, Gazyakan E, Bigdeli AK, Will-Marks P, Kneser U, Hirche C (2019). Soft tissue free flap for reconstruction of upper extremities: a meta-analysis on outcome and safety. Microsurgery..

[4] Li RG, Ren GH, Tan XJ, Yu B, Hu JJ (2013). Free flap transplantation combined with skin grafting and vacuum sealing drainage for repair of circumferential or sub-circumferential soft-tissue wounds of the lower leg. Med Sci Monit.

[5] Li RG, Yu B, Wang G, Chen B, Qin CH, Guo G (2012). Sequential therapy of vacuum sealing drainage and free-flap transplantation for children with extensive soft-tissue defects below the knee in the extremities. Injury..

[6] Franke A, Hentsch S, Bieler D, Schilling T, Weber W, Johann M (2017). Management of soft-tissue and bone defects in a local population: plastic and reconstructive surgery in a deployed military setting. Mil Med.

[7] Zheng X, Zheng C, Wang B, Qiu Y, Zhang Z, Li H (2016). Reconstruction of complex soft-tissue defects in the extremities with chimeric anterolateral thigh perforator flap. Int J Surg.

[8] Spyropoulou A, Jeng SF (2010). Microsurgical coverage reconstruction in upper and lower extremities. Semin Plast Surg.

[9] Huang HJ, Niu XH, Yang GL, Wang LY, Shi FC, Xu SJ, et al. Clinical effects of application of antibiotic bone cement in wounds of diabetic foot ulcers. *Zhonghua shao shang za zhi=Zhonghua shaoshang zazhi =* . Chin J Burns. 2019;35:464–6. 10.3760/cma.j.issn.1009-2587.2019.06.013 31280542

[10] Gage MJ, Yoon RS, Egol KA, Liporace FA (2015). Uses of negative pressure wound therapy in orthopedic trauma. Orthop Clin North Am.

[11] Liu X, Liang J, Zao J, Quan L, Jia X, Li M (2016). Vacuum sealing drainage treatment combined with antibiotic-impregnated bone cement for treatment of soft tissue defects and infection. Med Sci Monit.

[12] Numajiri T, Fau SY, Nishino K, Fau NK, Sugimoto K, Fau SK, Iwashina Y, Fau IY, Ikebuchi K, Fau IK, Nakano H (2012). Successful retrograde arterial inflow through a muscular branch in a free anterolateral thigh chimeric flap transfer. Microsurgery.

[13] Gao-Hong R, Run-Guang L, Gui-Yong J, Chao-Jie C, Zhi-Gang B (2017). A solution to the vessel shortage during free vascularized fibular grafting for reconstructing infected bone defects of the femur: Bridging with vein transplantation. Injury..

[14] Luo S, Raffoul W, Luo J, Luo L, Gao J, Chen L (1999). Anterolateral thigh flap: a review of 168 cases. Microsurgery..

[15] Lee YC, Chen WC, Chou TM, Shieh SJ (2015). Anatomical variability of the anterolateral thigh flap perforators: vascular anatomy and its clinical implications. Plast Reconstr Surg.

[16] Echo A, Bullocks JM (2011). Use of the descending branch of the lateral femoral circumflex vessels as a composite interposition graft in lower extremity reconstruction. Microsurgery..

[17] Qing L, Wu P, Yu F, Zhou Z, Tang J (2020). Use of a sequential chimeric perforator flap for one-stage reconstruction of complex soft tissue defects of the extremities. Microsurgery..

[18] Hallock GG (2011). The complete nomenclature for combined perforator flaps. Plast Reconstr Surg.

[19] Cherubino M, Turri-Zanoni M, Battaglia P, Giudice M, Pellegatta I, Tamborini F (2017). Chimeric anterolateral thigh free flap for reconstruction of complex cranio-orbito-facial defects after skull base cancers resection. J Craniomaxillofac Surg.

[20] Posch NA, Mureau MA, Flood SJ, Hofer SO (2005). The combined free partial vastus lateralis with anterolateral thigh perforator flap reconstruction of extensive composite defects. Br J Plast Surg.

[21] Qing L, Wu P, Zhou Z, Yu F, Tang J (2019). Customized reconstruction of complex three-dimensional defects in the extremities with individual design of vastus lateralis muscle-chimeric multi-lobed anterolateral thigh perforator flap. J Plast Surg Hand Surg.

[22] Zheng X, Zhan Y, Li H, Zhang Z, Xue X, Wang B (2019). Emergency repair of severe limb injuries with free flow-through chimeric anterolateral thigh perforator flap. Ann Plast Surg.

[23] Buono P, Castus P, Dubois-Ferriere V, Ruegg EM, Uckay I, Assal M, et al. Muscular versus non-muscular free flaps forsoft tissue coverage of chronic tibial osteomyelitis. World journal of plastic surgery 2018;7:294-300. 10.29252/wjps.7.3.294 PMC6290312 30560067

[24] Mohlhenrich SC, Kniha K, Elvers D, Ayoub N, Goloborodko E, Holzle F (2016). Intraosseous stability of dental implants in free revascularized fibula and iliac crest bone flaps. J Craniomaxillofac Surgery.

[25] Li R, Zhu G, Chen C, Chen Y, Ren G (2020). Bone transport for treatment of traumatic composite tibial bone and soft tissue defects: any specific needs besides the Ilizarov technique?. BioMed Res Int..

[26] Morelli I, Drago L, George DA, Gallazzi E, Scarponi S, Romano CL (2016). Masquelet technique: myth or reality? A systematic review and meta-analysis. Injury..

[27] Lee CH, Han SK, Dhong ES, Kim HP, Kim WK (2005). The fate of microanastomosed digital arteries after successful replantation. Plast Reconstr Surg.

